# Floodplain inundation in the Murray–Darling Basin under current and future climate conditions

**DOI:** 10.1038/s41598-025-93670-6

**Published:** 2025-03-14

**Authors:** Jin Teng, Francis H. S. Chiew, Hongxing Zheng, Ang Yang, David J. Penton, Catherine Ticehurst, Steve Marvanek, Jai Vaze, Fathaha Khanam, David A. Post, Carmel Pollino

**Affiliations:** 1 Commonwealth Scientific and Industrial Research Organization, Canberra, 2601 Australia; 2 Murray-Darling Basin Authority, Canberra, 2601 Australia

**Keywords:** Environmental water, Climate change, Floodplain management, Murray–Darling Basin, Climate sciences, Environmental sciences, Hydrology, Natural hazards

## Abstract

Predicting floodplain inundation under a changing climate is essential for adaptive management of water resources and ecosystems worldwide. This study presents a framework combining satellite observations and hydrological modeling to explore changes in floodplain inundation. We examine variability, trends, and frequency of inundation across the Murray–Darling Basin (MDB), Australia’s largest river system, over the past 35 years (1988–2022). Our analysis shows that annual maximum 30-day runoff is a primary hydrological factor influencing floodplain inundation. Using this metric as a proxy, we found that floodplain inundation, if driven solely by hydroclimate conditions, would have been more frequent in the recent decades (1988–2022) compared to the century-long baseline (1900–2022), especially in the southern basin. Despite projected declines in water availability under climate change in MDB, floodplain inundation appears to be less affected. The projected changes in floodplain inundation vary by region, influenced by local hydroclimate, human intervention, and the balance between projected more intense extreme rainfall and drier catchment conditions. This framework provides valuable insights into water resource planning and environmental management, with potential applications beyond the MDB.

## Introduction

Floodplain inundation plays a vital role in maintaining healthy ecosystems, as periodic flooding supports essential functions such as nutrient cycling and biodiversity preservation^1–3^. Climate change is expected to alter the intensity, frequency, timing, and extent of riverine floods^4,5^, although the impact varies regionally across the globe^6,7^. Stakeholders in agricultural, environmental, water resource, and other sectors are seeking insights into opportunities and risks from floodplain inundation, especially in the context of a changing climate^8–12^.

**Fig. 1:**
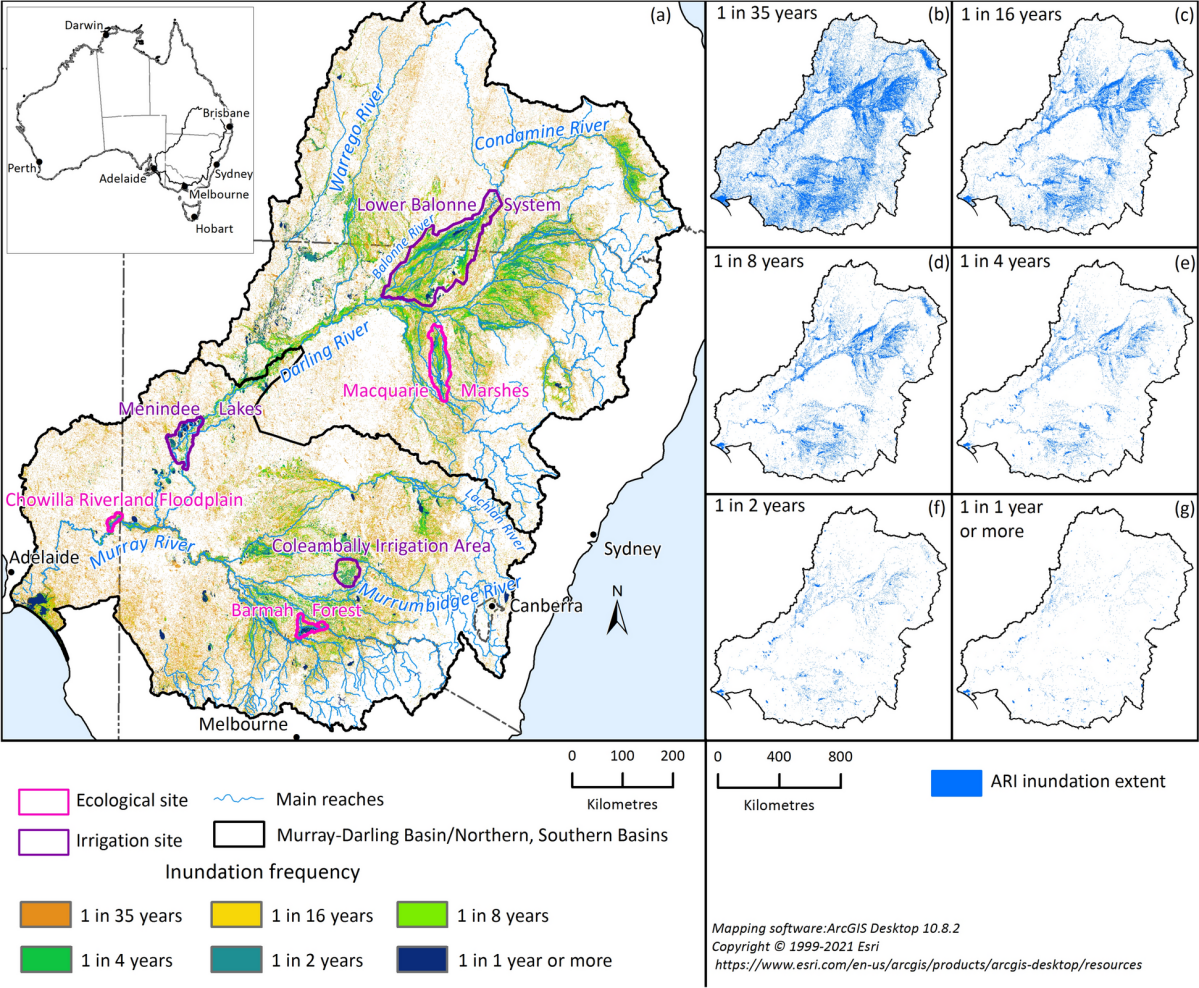
Floodplain inundation recurrence maps of the MDB. The frequency of surface water observed in the past 35 years. The flood extents for the various return periods are shown in separate panels (**b–g)** on the right. The main map (**a**) also shows the locations of the three irrigation sites and the three ecological sites.

The Murray–Darling Basin (MDB) (Fig. 1) is Australia’s largest, most economically significant, and politically complex river system^13^. With an agricultural industry worth AUD$24 billion annually, the MDB sustains 2.6 million people living in diverse rural and urban communities and supports vital environmental assets, including 16 Ramsar listed wetlands^14^. In the MDB, there is clear evidence of increasing precipitation extremes^15–17^; however, the consequences of flooding incidents are more nuanced^18,19^. The frequency of small and moderate floods in the MDB and southeastern Australia appears to be decreasing, while the magnitude of extreme floods is increasing^20^. Potential and contradicting explanations for this include more intense extreme rainfall^21^, reduced antecedent soil moisture^22,23^, shrinking storm coverage^24^, and decreased snowmelt^25^. Understanding the connection between shifting climate patterns and changes in flood dynamics presents a considerable challenge in the MDB. Floodplain inundation is generally considered beneficial in regional MDB due to its role in replenishing soil moisture, supporting biodiversity, and maintaining wetlands. Additionally, it can enhance water quality by flushing out accumulated nutrients and contaminants, providing vital ecosystem services.

Here, we developed a framework to investigate changes in flood inundation across the MDB for both the historical (1900–2022) and the future (2046–2075) periods by incorporating satellite observations, hydrological modeling, and climate change projections. We created inundation recurrence maps across the MDB using high-resolution Landsat observations^26,27^ (~ 30 m) spanning the last 35 years (1988–2022) and analyzed trends in inundation extent and volume during this period. To assess the changes in flood inundation beyond this observation period, we identified the dominant hydroclimate driver most closely correlated with inundation and used it as a proxy to assess changes in the probability of annual maximum flood inundation, measured as annual exceedance probabilities (AEP). We compared the AEP of the dominant hydroclimate driver, derived from hydrological modeling, over the past 35 years against its century-long baseline (1900–2022) as well as under future climate conditions informed by projections from the Coupled Model Intercomparison Project (phase 6) (CMIP6) general circulation models (GCMs).

To consider the spatial heterogeneity of flood inundation, we separated the MDB into the northern MDB and the southern MDB (see Fig. 1 for boundaries). The two regions have substantial differences in their hydrology, climate, land use, and water management practices. The northern MDB experiences higher seasonal and interannual hydroclimate variability, with more erratic rainfall patterns and drier conditions compared to the southern MDB. Most of the runoff originates from the high-elevation areas in the southeast of the basin, where most large reservoirs are located. In contrast, the northwest and west are arid, with long ephemeral and intermittent rivers flowing through relatively flatter landscapes^28,29^.

We also analyzed the results for three regulated areas—Coleambally Irrigation Area (CIA), Lower Balonne System (LBS), and Menindee Lakes (Menindee)—where river operators manage water storage and releases to meet surrounding and downstream irrigation demands (referred to as irrigation sites hereafter). Additionally, we examined three ecologically significant areas—Barmah Forest (Barmah), Chowilla Riverland Floodplain (Chowilla), and Macquarie Marshes (Macquarie)—which sustain diverse ecosystems, provide critical habitats, and support high levels of ecological productivity (referred to as ecological sites; see Fig. 1 for locations).

Each of these sites holds unique significance in terms of ecological value, human influence, and environmental challenges. The Coleambally Irrigation Area is one of the most productive irrigation districts in the MDB, where water management plays a crucial role in sustaining agricultural output. Similarly, the Lower Balonne System supports large-scale irrigation, which is facing significant pressures from climate change, over-extraction of water, and altered flow regimes. The Menindee Lakes, a series of naturally occurring and man-made lakes, function as critical water storage for both irrigation and environmental flows but have been subject to water losses due to evaporation and declining inflows in recent years^30^.

The Barmah Forest is one of the largest river red gum forests in the world, relying on periodic flooding to maintain its wetland ecosystem and support species such as waterbirds and native fish. The Chowilla Riverland Floodplain is a vital floodplain-wetland complex, playing a key role in maintaining water quality and biodiversity in the lower Murray region, yet it faces challenges from altered flow regimes and salinity^31^. The Macquarie Marshes, one of the largest inland wetlands in Australia, are highly dependent on natural flooding cycles for their extensive wetland habitat, which supports water dependent bird populations and diverse aquatic life^32^.

By selecting these contrasting sites, we aim to capture a broad spectrum of floodplain dynamics to provide insights on both the benefits and adverse effects of flooding across diverse environments.

## Results

### Flood-affected areas across the MDB

Flooding across the MDB for six average recurrence intervals (ARIs) are shown in Fig. 1b–g. The ARIs are calculated using 210 images (every 2 months over 35 years)^33^ derived from Landsat satellite observations. The two-monthly maximum water extent dataset was generated using all available images collected within each two-month period. These inundation recurrence maps offer insights not only into the locations of flood-prone areas but also into basin-wide hydrological connectivity, potential ecological hotspots, and major flood-dependent habitats. In the MDB, the pixels inundated at least once in the past 35 years covers approximately 274,049 km^2^, representing 25.9% of the total MDB area^25^. The area with a high inundation frequency (defined as an ARI ≤ 2 years) encompasses approximately 30,400 km^2^ (2.9%). The area with a medium inundation frequency (defined as an ARI between 8 and 16 years) covers approximately 79,204 km^2^ (7.5%).

For the three irrigation sites within the MDB, namely, the Coleambally Irrigation Area (CIA), Lower Balonne System (LBS), and Menindee Lakes (Menindee), the proportions of areas with high inundation frequency (ARI ≤ 2 years) are 14.8%, 11.5%, and 20.7%, respectively. For the three ecological sites, namely, Barmah Forest (Barmah), Chowilla Riverland Floodplain (Chowilla), and Macquarie Marshes (Macquarie), the corresponding proportions are 25.8%, 17.9%, and 8.1%, respectively. All these sites have areas with high inundation frequency above the basin average, with ecological sites being more prominent, reflecting the flood-dependent nature of these ecosystems.

### Trend and variation over the past 35 years

**Fig. 2:**
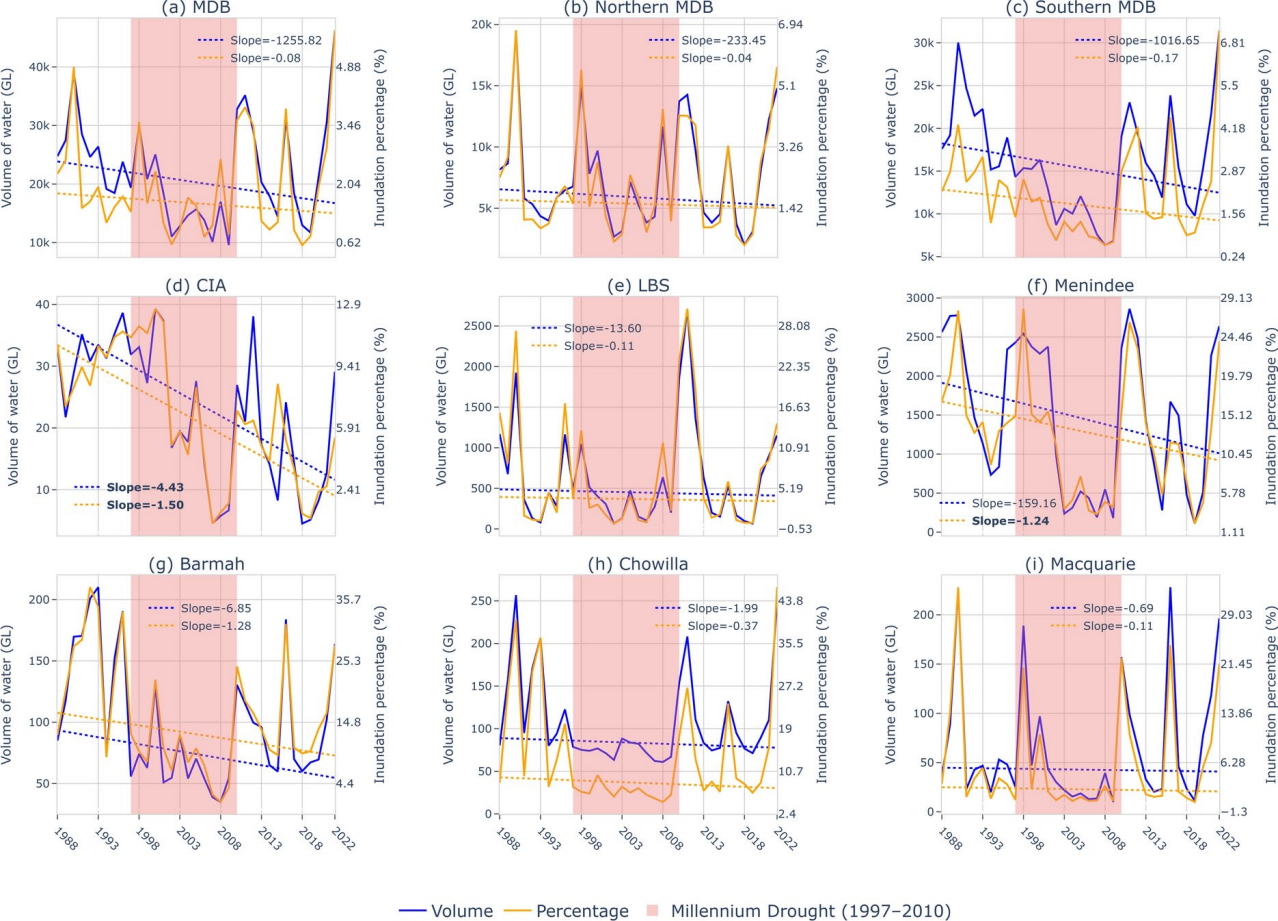
Annual maximum flood extent (orange) and volume (blue) for each region. The trends (GL per year or % per year), measured by Sen’s slope^45^, are shown on the top right and as trendlines in each plot. The bold font indicates that the trend is statistically significant at α < 0.05.

The variations in water extent and volume and trend over the past 35 years are presented in Fig. 2. The water extent is expressed as the percentage of inundated area relative to the total area in the regions considered. The volume is computed as the sum of the depth multiplied by the surface area for each pixel and is presented in Gigalitres (GL or 10^6^ m^3^). This analysis covers the entire MDB, northern MDB and southern MDB, and the three irrigation sites (CIA, LBS, and Menindee) and three ecological sites (Barmah, Chowilla, and Macquarie).

The analysis indicates that flooding magnitude is significantly influenced by extreme conditions, such as the Millennium Drought (1997–2010) and major floods in 2011 and 2022 (Fig. 2). There is a general declining trend in the annual maximum extent and volume of floodplain inundation across the MDB, which is more pronounced in the southern MDB (Fig. 2b) than in the northern MDB (Fig. 2c). However, it is important to note that these trends are not statistically significant due to high interannual variability in the basin. The high interannual hydroclimate variability in the MDB is primarily driven by climate drivers such as the El Niño-Southern Oscillation (ENSO), Indian Ocean Dipole (IOD), and Southern Annular Mode, which influence rainfall and temperature patterns^34^. Streamflow in the MDB, and in Australia generally, is more variable than rivers in similar hydroclimate areas elsewhere in the world^35,36^. Climate change and oceanic circulations also contribute to long-term variability, exacerbating droughts and floods in the region.

There is considerable variability in the extent of inundation both seasonally and annually across the MDB, reflecting the region’s high hydroclimate variability^37^. The intra-annual or seasonal variation, quantified by the maximum-minimum ratio (MV) of the inundation extent, is 2.85, 4.81, and 2.45 for the MDB, northern MDB, and southern MDB, respectively. The interannual variability is high, with coefficients of variation (CVs) of annual maximum inundation extent of 0.64, 0.76, and 0.64, in the MDB, northern MDB and southern MDB respectively (see Table S1 in the Supplementary Materials). The northern MDB exhibits greater seasonal and annual variability in flood-affected areas due to the strong influence of climate drivers such as ENSO and IOD, which create pronounced wet and dry cycles^34^. Additionally, the region is more arid, characterized by ephemeral rivers, a lower runoff coefficient, and significant runoff driven by fewer intermittent rainfall events^30,38^.

For the three irrigation sites, the intra-annual variability (MV) in the CIA, LBS, and Menindee are 19.34, 2.95, and 8.42, respectively. The interannual CVs of the maximum inundation extent are 0.56, 1.18, and 0.56, while the CVs of the minimum inundation extent are 0.42, 1.64, and 0.69, respectively. CIA displays significant declining trend in both inundation extent and floodwater volume, with rates of − 1.50% per year and − 4.43 GL per year, respectively. Before the mid-2000s, the regular inundation patterns in the CIA resembled the seasonal flood irrigation used for rice paddocks (see Fig. S1 in the Supplementary Materials). After the mid-2000s, the shift to less regular inundation coincided with the adoption of more efficient irrigation infrastructure and changes in crop types in the area^39,40^. Another site that has a statistically significant declining trend in flood extent is Menindee, which has experienced mass fish kills in recent years^41–43^.

The dynamics of inundation at the three ecological sites differ from those at the irrigation sites, characterized by relatively lower variations and less significant trends, likely due to less development and environmental water delivery since 2012^44^. The intra-annual variability (MV) is 7.79 for Barmah, 2.48 for Chowilla, and 25.79 for Macquarie. Correspondingly, the interannual variability (CV) in the maximum inundation extent is 0.61 for Barmah, 0.81 for Chowilla, and 1.37 for Macquarie, while the CVs in the minimum inundation extent are 0.46, 0.22, and 0.69, respectively. These differences highlight the unique hydrological characteristics of ecological sites, which are influenced by natural variability and environmental water management practices.

The comparison between irrigation and ecological sites reveals both similarities and differences in their intra- and interannual variability. Irrigation sites, such as CIA, LBS, and Menindee, tend to show lower intra-annual variability (MV) in inundation extent, particularly the LBS and Chowilla, suggesting more controlled and stable conditions. However, the ecological sites, such as Barmah, Chowilla, and Macquarie, generally exhibit higher interannual variability in maximum and minimum inundation extent, especially Macquarie. While the CVs for maximum inundation extent are more variable in ecological sites (ranging from 0.61 to 1.37), the irrigation sites show more consistency (ranging from 0.56 to 1.18), highlighting that ecological sites experience more fluctuation due to their reliance on natural flood dynamics. Additionally, ecological sites like Macquarie exhibit greater intra-annual variability in inundation extent (MV of 25.79), reflecting more dynamic floodplain conditions and stronger dependency on seasonal flood events compared to irrigation sites with more regulated environments.

### Dominant hydroclimate drivers of floodplain inundation

**Fig. 3:**
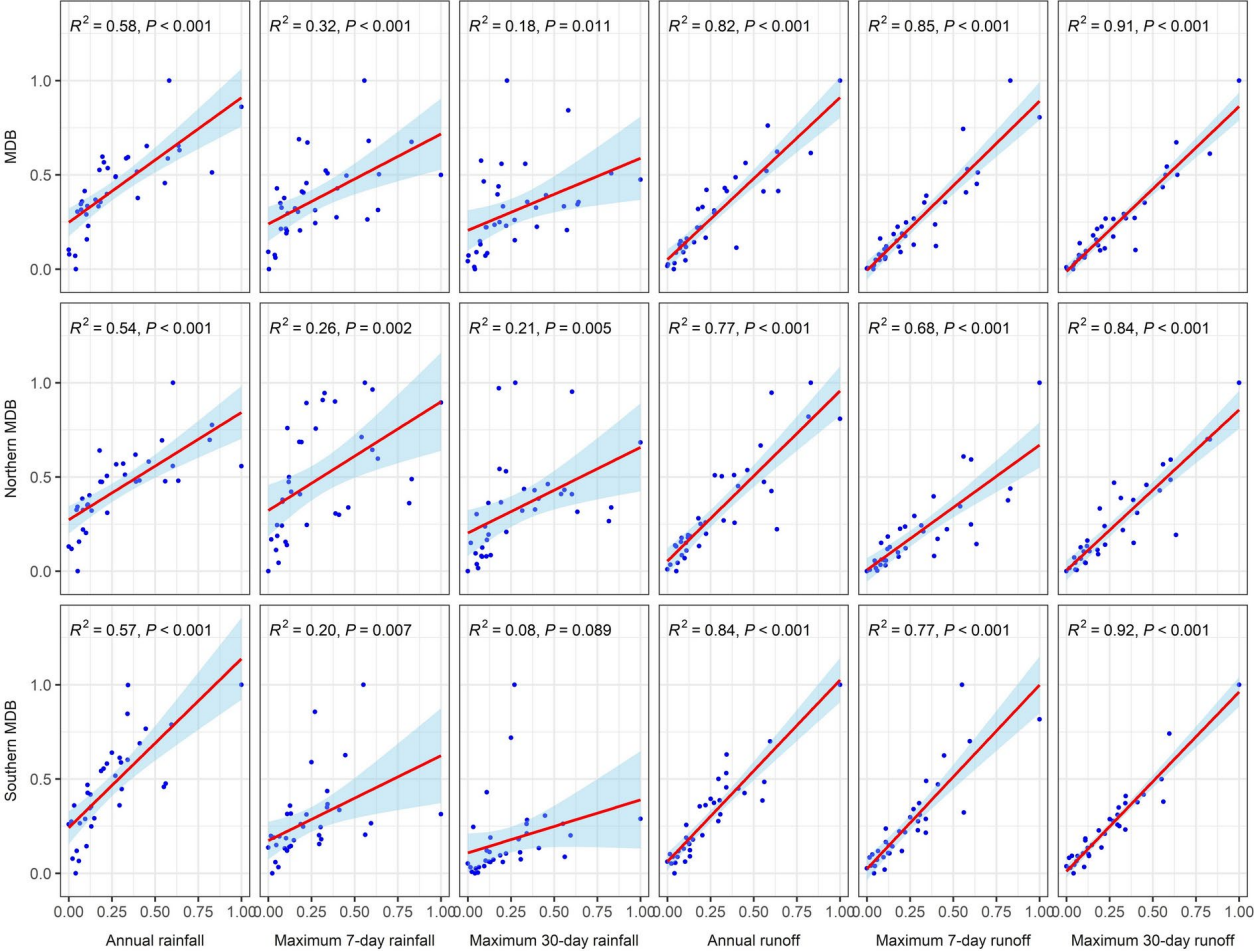
Correlation between hydroclimatic variables and annual maximum inundation extent. The *x*-axis shows the hydroclimatic variables, and the *y*-axis shows the maximum inundation extent, both scaled to 0–1 based on minimum and maximum values in the data.

Floodplain inundation is influenced by hydroclimatic conditions such as preceding rainfall and streamflow. Identifying the dominant drivers can help predict how climate change might affect floodplain inundation. Figure 3 and Table S2 in the Supplementary Materials show the correlations of various hydroclimate variables with the annual maximum floodwater extent. The choice of these hydroclimate variables is guided by insights from a previous study^46^. The rainfall variables are derived from observations, while the runoff variables are results from hydrological modeling (see the Method section for details).

For all the regions considered here, the maximum 30-day runoff has the strongest correlation with the inundation extent and water volume, followed by the annual runoff and the maximum 7-day runoff. Therefore, maximum 30-day runoff is used as a proxy for the annual maximum floodplain extent.

Correlations between dominant hydroclimate variables and inundation extent are stronger in the southern MDB than in the northern MDB. The stronger inundation response of the southern MDB to the hydroclimate is likely due to the higher runoff volumes and lower intra- and interannual variability compared to those in the northern basin.

Overall, the three ecological sites demonstrate greater sensitivity to hydroclimate conditions than the irrigation sites. This is likely due to changes in the relationship between inundation and hydroclimate conditions resulting from development and practice over time at irrigation sites, particularly at the CIA.

### Floodplain inundation under a changing climate

**Fig. 4:**
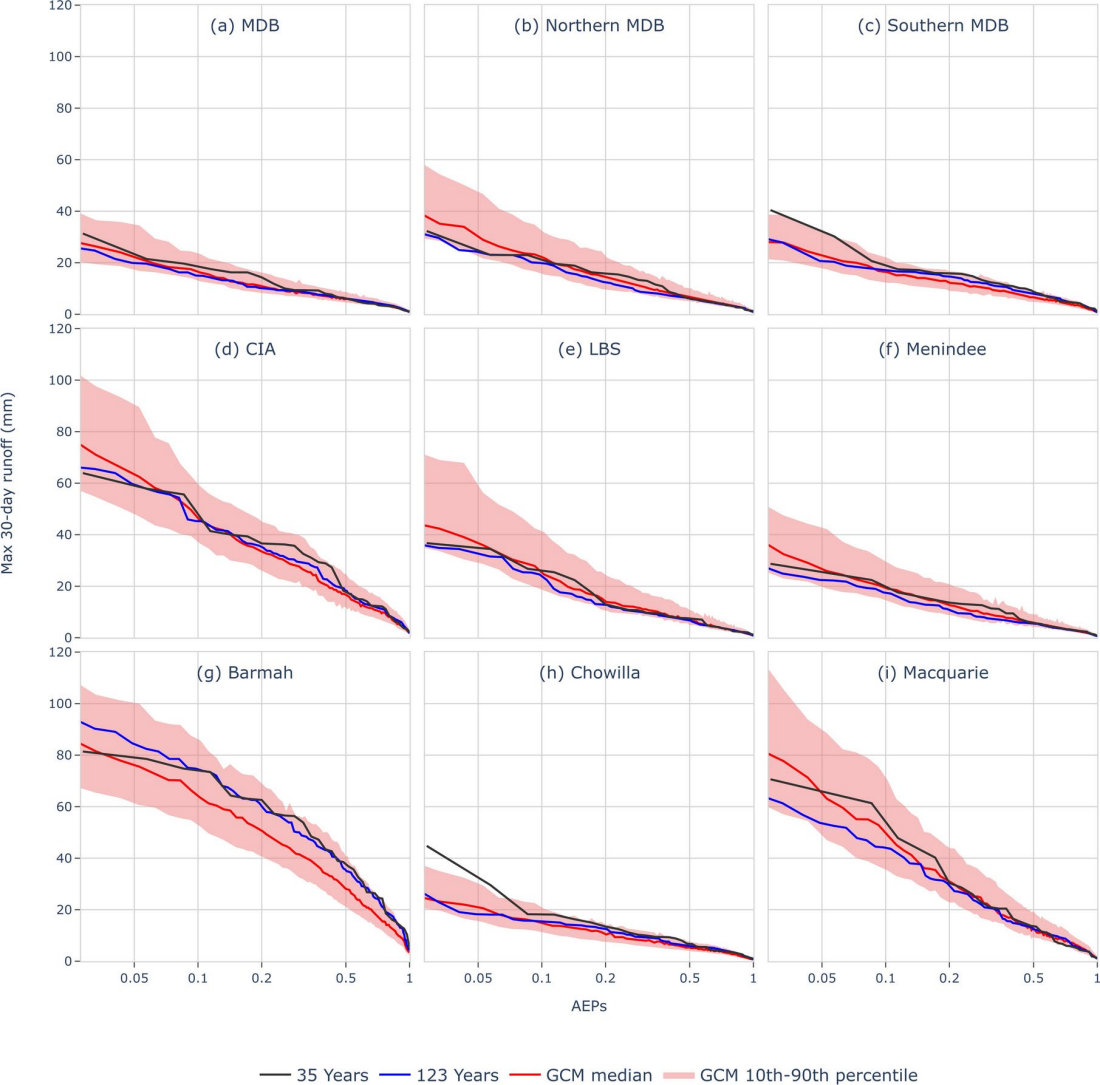
Comparison of recent, historical, and future floodplain inundation using a proxy. AEPs for 35 recent observational years (1988–2022), 123 historical years (1900–2022), and future projections (2024–2075), where an AEP of 0.05 is equivalent to an ARI (= 1/AEP) of 20 years of floodplain inundation.

Further investigation reveals shifts in the magnitude and frequency of the annual maximum inundation extent under climate change using the maximum 30-day runoff as a proxy. The annual exceedance probabilities (AEPs) of maximum 30-day runoff in the recent period (1988–2022) are shown to be greater than in the long-term baseline (1900–2022) (Fig. 4).

As indicated by the proxy, the higher inundation frequency in the last 35 years is more prominent in the southern MDB (Fig. 4c). This is particularly evident for more extreme floodplain inundation events (AEP < 0.1 or ARI > 10 years), consistent with the results of previous studies^15,20^. In the northern MDB, inundation frequency in the last 35 years is generally similar to, or only slightly greater than, the 123-year baseline period (Fig. 4b).

As shown by previous studies, future hydroclimate projections indicate a drier MDB, with significantly lower mean annual runoff driven by reductions in cool-season rainfall and increased potential evaporation^47^. However, floodplain inundation, as represented by the proxy, appears to be less affected by climate change than mean annual runoff (Fig. 4 red lines vs. blue lines). This is particularly evident in the northern MDB, where moderate to extreme flood events (AEP < 0.1) are even projected to increase under future climate conditions (Fig. 4b). This increase can be attributed to the intensification of extreme rainfall, which more than compensates for drier antecedent catchment conditions. The impact is especially pronounced in the northern MDB, where floods are primarily generated by a small number of intermittent events, and the projected decline in rainfall and runoff is less significant compared to the perennial systems in the southern MDB. In the southern MDB (Fig. 4c), rarer flood events (AEP < 0.1) are projected to remain similar to the baseline, while more frequent floodplain inundation events (AEP > 0.1, or occurring on average once every ten years) are expected to decrease under climate change. However, this decline is smaller than the projected reduction in mean annual runoff^48–50^.

**Fig. 5:**
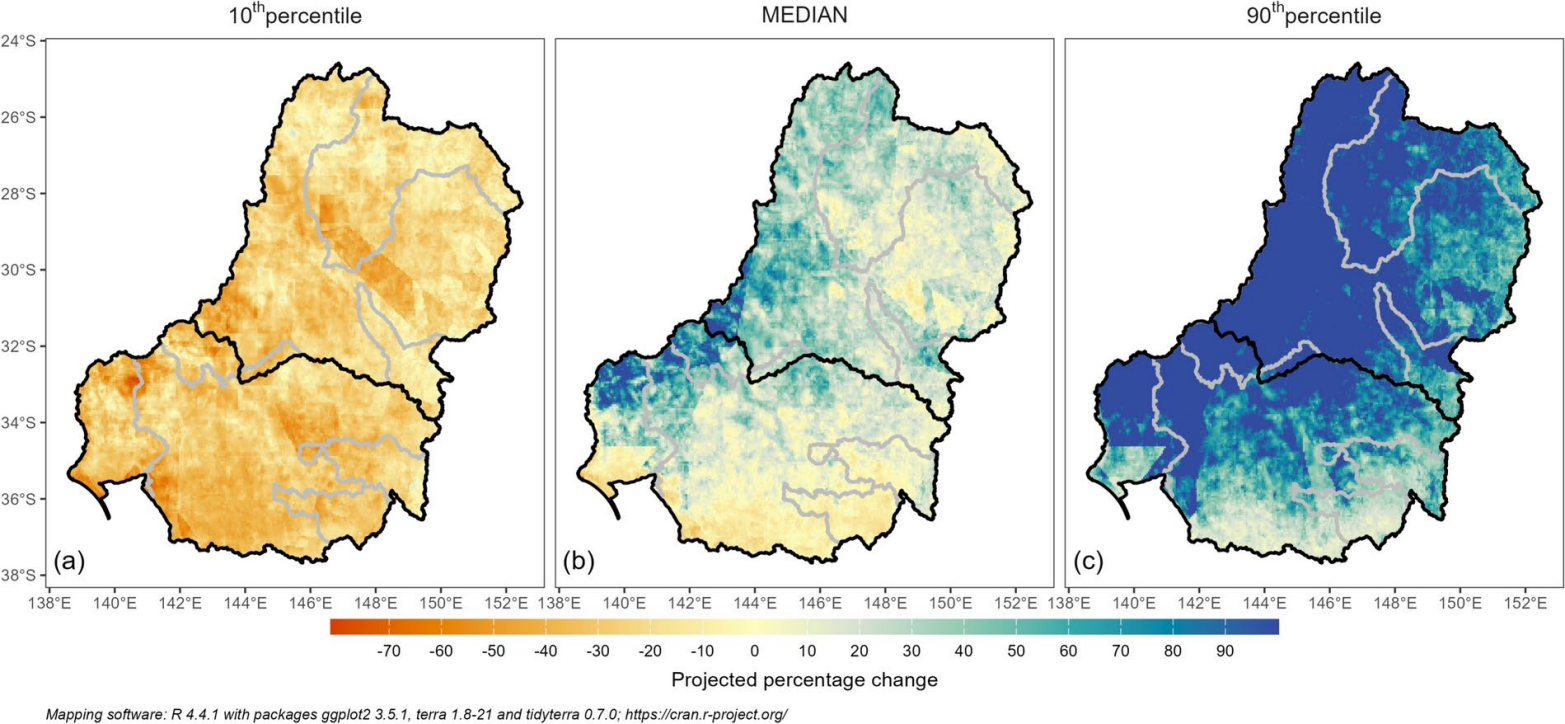
Projected percentage change in maximum 30-day runoff with an ARI of 20 years. The baseline period of the projection is 1900–2022, and the future period is 2046–2075. The 10th percentile, median and 90^th^ percentile are obtained from simulations informed by 37 GCMs (see Methods). The gray boundaries are upstream contributing areas of the six irrigation and ecological sites. The black boundary separates the northern and southern MDB.

Figure 5 shows the projected changes in the magnitude of 1-in-20-year floodplain inundation events (AEP = 0.05) represented by the maximum 30-day runoff proxy under future climate simulations informed by 37 CMIP6 GCMs (see Methods). In general, the median projection (Fig. 5b) shows an increase in 1-in-20-year floodplain inundation (in the annual maximum 30-day runoff proxy) across the dry areas in the northwest and western parts of the Basin, and a small decrease in the far south of the Basin^39^. Nevertheless, there is large uncertainty in the projections due to the large range in the GCM rainfall projections (Fig. 5).

For the six irrigation and ecological sites, an intensification of 1-in-20-year floodplain inundation is projected at all sites except Barmah. This exception is due to Barmah’s southernmost location, where the significant decline in cool-season rainfall results in drier antecedent catchment conditions, outweighing the effects of increased extreme rainfall. Conversely, a marked increase in 1-in-20-year floodplain inundation is projected for Macquarie, a site located in the northern MDB. This underscores the need for region-specific water management strategies, particularly in areas like Barmah and Macquarie.

## Discussion

The knowledge from this study has significant implications for communities, decision-makers, environmental watering, and stakeholders in the MDB. We found that the maximum 30-day runoff closely correlated with floodplain inundation in the MDB. This finding offers a reliable proxy for floodplain inundation, which is useful for applications such as land-use planning, habitat restoration, and water resource management, particularly given the scarcity of reliable spatial inundation data.

Future runoff in the MDB is likely to decrease under climate change due to projected reduction in cool season rainfall and accentuated by higher potential evaporation^51^. This outlook is supported by multiple lines of evidence, such as trends in observed data, projections from climate models, and changes in global and regional circulation patterns under higher temperature^52^. A reduction in runoff, coupled with increasing water demand from many uses, will intensify the already fierce competition for water^53,54^. On a positive note, the analysis here suggests that the impact of climate change on floodplain inundation is likely to be less severe than its impact on water resources and hydrological droughts. This is because the effects of intensifying extreme rainfall and decreasing antecedent catchment conditions offset each other. Floodplain inundation frequency in the northern MDB may remain unchanged or even increase for rarer, more extreme events (see Section "Floodplain inundation under a changing climate"). In the southern MDB, rarer events are expected to stay similar to the baseline, while more frequent inundation events are projected to decline under climate change, though to a lesser extent than mean annual runoff.

Results also show that floodplain inundation over the past 35 years would have been greater and more frequent than the long historical baseline if driven solely by hydroclimate conditions. Despite this, significant challenges in water resource management have affected floodplain outcomes across the basin^55–58^. Over-extraction and altered flow regimes have contributed to floodplain degradation, declines in vegetation and animal species, the spread of invasive species and water quality issues. These challenges emphasize the urgent need for adaptive management strategies to improve floodplain outcomes, rather than attributing the impacts mainly to climate change^59–61^.

One key user of water in the MDB is the Commonwealth Environment Water Holder (CEWH), which buys and uses water to enhance environmental outcomes^62^. Data on current and future floodplain inundations are vital to their operations. The projected future flooding varies from region to region, highlighting the need for tailored decision-making. For example, the Barmah Forest in the Southern MDB and the Macquarie Marshes in the Northern MDB, both of which rely on periodic flooding to sustain floodplains and wetlands, are projected to experience different changes; Barmah is likely to experience less flooding, while Macquarie may experience more flooding. The quantification of changes in this study can assist water managers such as CEWH in developing customized environmental water delivery plans to address specific challenges for different locations.

The outcome of this study can also help identify knowledge gaps and priorities for research. We found significant regional variations in the flood extent and volume trends in recent years. Some areas, such as the Menindee Lakes, show statistically significant reductions in flooding, whereas nearby Chowilla Riverland Floodplains do not show the same trend. The underlying causes of these differences, as well as their ecological and social implications, remain unclear. Potential contributing factors include high temperatures increasing evaporation, overextraction of water in the Menindee Lakes, the delivery of environmental water in Chowilla, or a combination of these influences. Quantifying the impact of these factors can inform policy adjustments to better balance ecological sustainability and agricultural demands. The decrease in flooding at highly managed sites such as the CIA likely reflects the effectiveness of new regulations and the modernization of irrigation practices, resulting in more efficient water use. Additionally, the shift in crop types from rice to cotton and maize may have contributed to changes in water management and inundation patterns^40^. Further research is needed to understand the linkage between water allocation, farming practice, and inundation patterns, as well as their long-term impacts. This knowledge can help optimize water allocation strategies for sustainable management.

For researchers globally, the methodology presented here offers valuable insights into the evolving nature of floodplain inundation influenced by water regulation and climate change. The analysis demonstrated that changes in flooding can vary across regions and are influenced by diverse factors, such as spatially heterogeneous rainfall patterns, irrigation practices, and climate change. Recognizing these differences is essential when engaging with policy makers, environmental managers, communities and stakeholders. Through the development of comprehensive spatial flooding datasets, hydrological modeling and climate change projections, researchers can help stakeholders respond to similar challenges worldwide.

## Methods

This study presents the observed frequency, variability and trend of floodplain inundation across the MDB from 1988 to 2022 and characterizes floodplain inundation for the historical period (1900–2022) and projected future under climate change. A consistent framework is applied across the MDB, leveraging on remote sensing and hydrological modeling to analyze the spatial and temporal characteristics of floodplain inundation under historical, recent, and future climate conditions. While previous studies utilizing remote sensing data primarily focused on mapping inundation dynamics^63–66^, wetlands^67^, or human impacts^68^ within observed time frames, this study builds on that foundation by incorporating both historical and projected hydroclimate data to assess floodplain inundation under a changing climate.

### Satellite-based floodplain inundation observation

The two-monthly maximum floodwater depth dataset^33,69,70^ covers the period from 1988 to 2022 at a spatial resolution of approximately 30 m. This dataset provides a spatial representation of the maximum surface water extent and depth across the MDB at two-month intervals. Surface water was detected from Landsat imagery using the multi-index method (MIM)^71^ for enhanced accuracy. Water depth was estimated using an improved version of the Floodwater Depth Estimation Tool (FwDET)^72,73^, which takes water extent and a LiDAR-enhanced high-resolution Digital Elevation Model (DEM)^74^ as inputs. This dataset offers a comprehensive view of observed inundation extent and water depth across the MDB and reveals the historical lateral connectivity throughout the basin.

### Rainfall and potential evapotranspiration under future climate scenarios

For the historical period, daily rainfall of each grid cell was sourced from the SILO gridded dataset^75^, and potential evapotranspiration was calculated from the SILO climate surfaces using Morton’s wet environment algorithms^76,77^. Future rainfall projections were generated using the Daily Scaling method^78^, informed by climate change signals from 37 CMIP6 GCMs for Shared Socioeconomic Pathway 5–8.5 (SSP5-8.5) (for a 30-year time slice centered on 2060 relative to a 30-year time slice centered on 1990, representing approximately 2.3 °C global average warming)^79^. The daily scaling method perturbs historical climate time series data based on the change signals derived from GCMs, reflecting changes in both the means and the shape of the daily rainfall distribution, which is important for capturing the intensification of extreme heavy rainfall simulated by GCMs. Future potential evapotranspiration was generated using the seasonal scaling method^78^, which was also informed by the corresponding CMIP6 GCMs.

### Modeling historical and future runoff across the basin

Historical and future runoff were simulated for each 0.05° grid cell in the MDB using the GR4J model^80^. The GR4J model is a daily hydrological model with four parameters. The model was calibrated and validated against observed streamflow from 780 unimpaired headwater catchments across Australia, with a nearest neighbor approach applied in parameter regionalization^81,82^. For the historical period (1900–2022), daily runoff for each grid cell was modeled using daily rainfall and potential evapotranspiration from the SILO gridded dataset. For the future period (centered at approximately 2060 with the same length as the historical period), the model inputs were the scaled rainfall and potential evapotranspiration information obtained from the CMIP6 GCMs, as described in Rainfall and potential evapotranspiration under future climate scenarios. The model calibration, by maximizing the NSE-Bias (Nash–Sutcliffe Efficiency with constraint on Overall Bias) objective function^83^, provides reasonable simulations of median and high flows, including the maximum 30-day runoff analyzed in this study^28^.

### Defining the dominant hydroclimate drivers of floodplain inundation

With the SILO climate data and modeled daily runoff, we identified the dominant hydroclimate drivers of floodplain inundation by correlating candidate hydroclimate metrics against both the inundation extent and water volume at the annual scale. The metrics considered included annual rainfall and runoff, monthly rainfall and runoff, and the maximum n-day (n = 1, 3, 5, 7, 30) rainfall and runoff. The metric with the highest correlation coefficient was selected as the dominant hydroclimate driver and used as a proxy for floodplain inundation.

While correlation does not imply causation, our objective was to identify a practical and readily available metric that effectively represents floodplain inundation dynamics, rather than to establish a direct causal relationship. Floodplain inundation is influenced by various factors, including soil moisture, topography, and floodplain-channel connectivity. Although these factors were not explicitly modeled, they are indirectly reflected in the historical inundation data used to derive correlation. Given these considerations, the selected proxy serves as a reasonable indicator of large-scale inundation patterns across the MDB.

However, this approach does not explicitly account for streamflow lag or water management activities, such as the operation of reservoirs, pumps, and other physical structures that influence the timing and distribution of river flows. Additionally, while antecedent catchment conditions are crucial in shaping flood responses, they are only implicitly considered through the rainfall-runoff modeling.

### Limitations and uncertainty

There are limitations and uncertainties in the Landsat-derived water extent. Cloud cover and vegetation can obscure satellite imagery, leading to gaps or inaccuracies in detecting water extent. These challenges are compounded in forested or heavily vegetated floodplains, where water beneath the canopy may go undetected. Additionally, the revisit period of Landsat observations results in missed flood peaks, particularly for events with short durations, contributing to an overall underestimation of flood extent and depth. Moreover, our study primarily focused on flood intensity and frequency, without considering potential changes in flood duration, which could influence ecological functions as well as associated socio-economic and environmental impacts.

Hydrological modeling introduces further uncertainties due to the assumptions and simplifications inherent in representing complex natural systems^84^. The same parameter values are used to model historical and future runoff. The extrapolation of models developed and calibrated against historical data to predict the future is a significant limitation and challenge^85,86^. Factors such as inaccuracies in input data, calibration limitations, and the exclusion of human influences like dam operations or irrigation can lead to deviations between modeled and observed hydrological behaviour.

Further, this study is based on empirical evidence, which inherently includes human intervention that can significantly alter flood dynamics. However, we were unable to isolate the individual impacts of hydroclimate and human intervention on floodplain inundation due to the lack of long-term monitoring of land use changes, infrastructure modifications, and operational activities such as retention, diversion, and extraction.

Climate projections, while essential for understanding future flood dynamics, add another layer of uncertainty. Nevertheless, appropriate fit-for-purpose datasets and modeling are used here, in a consistent framework across the entire basin, enabling a consistent interpretation of spatial and temporal characteristics of floodplain inundation across the Basin under historical and future climate conditions.

## Supplementary Information

Supplementary Information.

## Data Availability

All the data presented in this paper is publicly available via the CSIRO Data Access Portal at 10.25919/ka7q-ne58.

## References

[1] Serra-Llobet, A. *et al.* Restoring Rivers and Floodplains for Habitat and Flood Risk Reduction: Experiences in Multi-Benefit Floodplain Management From California and Germany. *Front Environ Sci* **9**, (2022).

[2] Pratt, O. P., Beesley, L. S., Pusey, B. J., Setterfield, S. A. & Douglas, M. M. The implications of brief floodplain inundation for local and landscape-scale ecosystem function in an intermittent Australian river. *Mar Freshw Res* **75**, (2024).

[3] Opperman, J. J., Luster, R., McKenney, B. A., Roberts, M. & Meadows, A. W. Ecologically Functional Floodplains: Connectivity, Flow Regime, and Scale1. *JAWRA Journal of the American Water Resources Association* **46**, 211–226 (2010).

[4] Blöschl, G. et al. Changing climate shifts timing of European floods. *Science* **1979**(357), 588–590 (2017). 10.1126/science.aan2506 28798129

[5] Alifu, H., Hirabayashi, Y., Imada, Y. & Shiogama, H. Enhancement of river flooding due to global warming. *Sci Rep* **12**, (2022). 10.1038/s41598-022-25182-6 PMC9712344 36450837

[6] Blöschl, G. et al. Changing climate both increases and decreases European river floods. *Nature* **573**, 108–111 (2019). 31462777 10.1038/s41586-019-1495-6

[7] Gu, X. H. *et al.* The changing nature and projection of floods across Australia. *J Hydrol (Amst)* **584**, (2020).

[8] Schmocker-Fackel, P. & Naef, F. More frequent flooding? Changes in flood frequency in Switzerland since 1850. *J Hydrol (Amst)* **381**, 1–8 (2010).

[9] Smith, A., Freer, J., Bates, P. & Sampson, C. Comparing ensemble projections of flooding against flood estimation by continuous simulation. *J Hydrol (Amst)* **511**, 205–219 (2014).

[10] Chen, J. *et al.* Impacts of climate warming on global floods and their implication to current flood defense standards. *J Hydrol (Amst)* **618**, 129236 (2023).

[11] Di Baldassarre, G. Floods in a Changing Climate: Inundation Modelling. *Floods in a Changing Climate: Inundation Modelling*10.1017/CBO9781139088411 (2010).

[12] Douglas, E. M., Vogel, R. M. & Kroll, C. N. Trends in floods and low flows in the United States: impact of spatial correlation. *J Hydrol (Amst)* **240**, 90–105 (2000).

[13] Lawrence, J. *et al.* Australasia. in *Climate Change 2022 – Impacts, Adaptation and Vulnerability. Contribution of Working Group II to the Sixth Assessment Report of the Intergovernmental Panel on Climate Change* (eds. Pörtner, H.-O. et al.) 1581–1688 (Cambridge University Press, 2022). 10.1017/9781009325844.013.

[14] DAWE. Murray Darling Basin Plan. Preprint at https://www.agriculture.gov.au/water/mdb (2012).

[15] Wasko, C. & Sharma, A. Steeper temporal distribution of rain intensity at higher temperatures within Australian storms. *Nat Geosci* **8**, 527–529 (2015).

[16] Guerreiro, S. B. et al. Detection of continental-scale intensification of hourly rainfall extremes. *Nat Clim Chang* **8**(9), 803–807 (2018).

[17] Dowdy, A. J. et al. Review of Australian east coast low pressure systems and associated extremes. *Clim Dyn* **53**, 4887–4910 (2019).

[18] Sharma, A., Wasko, C. & Lettenmaier, D. P. If Precipitation Extremes Are Increasing, Why Aren’t Floods?. *Water Resour Res* **54**, 8545–8551 (2018).

[19] Bennett, B., Leonard, M., Deng, Y. & Westra, S. An empirical investigation into the effect of antecedent precipitation on flood volume. *J Hydrol (Amst)* **567**, 435–445 (2018).

[20] Wasko, C., Guo, D., Ho, M., Nathan, R. & Vogel, E. Diverging projections for flood and rainfall frequency curves. *J Hydrol (Amst)* **620**, 129403 (2023).

[21] Ho, M. *et al.* Changes in flood-associated rainfall losses under climate change. *J Hydrol (Amst)* **625**, 129950 (2023).

[22] Hettiarachchi, S., Wasko, C. & Sharma, A. Can antecedent moisture conditions modulate the increase in flood risk due to climate change in urban catchments?. *J Hydrol (Amst)* **571**, 11–20 (2019).

[23] Wasko, C. & Nathan, R. Influence of changes in rainfall and soil moisture on trends in flooding. *J Hydrol (Amst)* **575**, 432–441 (2019).

[24] Wasko, C., Sharma, A. & Westra, S. Reduced spatial extent of extreme storms at higher temperatures. *Geophys Res Lett* **43**, 4026–4032 (2016).

[25] Di Luca, A., Evans, J. P. & Ji, F. Australian snowpack in the NARCliM ensemble: evaluation, bias correction and future projections. *Clim. Dyn.* **51**, 639–666 (2018).

[26] Vermote, E., Justice, C., Claverie, M. & Franch, B. Preliminary analysis of the performance of the Landsat 8/OLI land surface reflectance product. *Remote Sens Environ* **185**, 46–56 (2016). 10.1016/j.rse.2016.04.008 PMC6999666 32020955

[27] Lewis, A. et al. The Australian Geoscience Data Cube — Foundations and lessons learned. *Remote Sens Environ* **202**, 276–292 (2017).

[28] Chiew, F. H. S., Zheng, H., Post, D. A., Robertson, D. E. & Rojas, R. *Hydroclimate Trends and Future Projections in the Murray-Darling Basin*. https://www.mdba.gov.au/sites/default/files/publications/mdb-outlook-hydroclimate-literature-review2.pdf (2022).

[29] Barry Hart, Neil Byron, Nick Bond, Carmel Pollino & Michael Stewardson. *Murray-Darling Basin, Australia: Its Future Management*. (Elsevier, 2020).

[30] MDBA. *The 2020 Basin Plan Evaluation*. https://www.mdba.gov.au/sites/default/files/pubs/bp-eval-2020-full-report.pdf (2020).

[31] MDBA. *Assessment of Environmental Water Requirements for the Proposed Basin Plan: Riverland–Chowilla Floodplain*. (2012).

[32] Roberts, J. & Marston, F. *Water Regime for Wetland and Floodplain Plants: A Source Book for the Murray-Darling Basin*. (Australian Government: National Water Commission, 2011).

[33] Ticehurst, C., Penton, D., Teng, J. & Sengupta, A. Maximum two-monthly surface water extent for MDB from MIM and WOFS - Version 2. *CSIRO. Data Collection* (2023) 10.25919/s7c2-hc39.

[34] Gallant, A. J. E., Kiem, A. S., Verdon-Kidd, D. C., Stone, R. C. & Karoly, D. J. Understanding hydroclimate processes in the Murray-Darling Basin for natural resources management. *Hydrol Earth Syst Sci* **16**, 2049–2068 (2012).

[35] Chiew, F. H. S. & McMahon, T. A. *Climate Variability, Climate Change and Water Resources in Australia*. *Proceedings of the Second International Conference on Climate and Water, Vols 1–3* (1998).

[36] Peel, M. C., McMahon, T. A. & Finlayson, B. L. Continental differences in the variability of annual runoff-update and reassessment. *J. Hydrol. (Amst.)* **295**, 185–197 (2004).

[37] Chiew, F. H. S. & McMahon, T. A. Global ENSO-streamflow teleconnection, streamflow forecasting and interannual variability. *Hydrol. Sci. J.* **47**, 505–522 (2002).

[38] Hart, B., Byron, N., Bond, N., Pollino, C. & Stewardson, M. *Murray-Darling Basin, Australia: Its Future Management* (Elsevier, 2020).

[39] Our Story—Coleambally Irrigation. https://www.colyirr.com.au/our-story.

[40] Murray-Darling Basin water markets: trends and drivers 2002–03 to 2018–19. https://daff.ent.sirsidynix.net.au/client/en_AU/ABARES/search/detailnonmodal/ent:$002f$002fSD_ASSET$002f0$002fSD_ASSET:1029942/one.

[41] Academy of Science, A. *Investigation of the Causes of Mass Fish Kills in the Menindee Region NSW over the Summer of 2018–2019*. www.science.org.au/fish-kills-report (2019).

[42] Vertessy, R. *et al. Final Report of the Independent Assessment of the 2018-19 Fish Deaths in the Lower Darling*. https://s3-ap-southeast-2.amazonaws.com/figshare-production-eu-latrobe-storage9079-ap-southeast-2/31186917/1185369_VertessyR_2019.pdf?X-Amz-Algorithm=AWS4-HMAC-SHA256&X-Amz-Credential=AKIARRFKZQ25KW2DIYRU/20250110/ap-southeast-2/s3/aws4_request&X-Amz-Date=20250110T060546Z&X-Amz-Expires=10&X-Amz-SignedHeaders=host&X-Amz-Signature=0303ca3c2d4f5f2d4e1e875b257e7083da480bd76ddebf8627b2fb927df249fa (2019).

[43] Jackson, S. & Head, L. Australia’s mass fish kills as a crisis of modern water: Understanding hydrosocial change in the Murray-Darling Basin. *Geoforum* **109**, 44–56 (2020).

[44] Murray–Darling Basin Authority. *The Murray–Darling Basin Authority Annual Report 2012–13* (2013).

[45] Sen, P. K. Estimates of the regression coefficient based on Kendall’s Tau. *J. Am. Stat. Assoc.* **63**, 1379–1389 (1968).

[46] Fu, G. et al. Statistical analysis of attributions of climatic characteristics to nonstationary rainfall-streamflow relationship. *J. Hydrol. (Amst.)* **603**, 127017 (2021).

[47] Prosser, I. P., Chiew, F. H. S. & Smith, M. S. Adapting water management to climate change in the Murray–Darling Basin, Australia. *Water (Switzerland)* **13**, 1–19 (2021).

[48] Speer, M. S., Leslie, L. M., MacNamara, S. & Hartigan, J. From the 1990s climate change has decreased cool season catchment precipitation reducing river heights in Australia’s southern Murray-Darling Basin. *Sci. Rep.* **11**(1), 1–16 (2021). 34373547 10.1038/s41598-021-95531-4 PMC8352959

[49] Golding, B. & Campbell, C. Learning to be drier in the southern Murray-Darling Basin: Setting the scene for this research volume. *Aust. J. Adult Learn.* **49**, 423–450 (2009).

[50] Potter, N. J. & Chiew, F. H. S. An investigation into changes in climate characteristics causing the recent very low runoff in the southern Murray-Darling Basin using rainfall-runoff models. *Water Resour. Res.* **47** (2011).

[51] Whetton, P. & Chiew, F. Climate change in the Murray-Darling Basin. In *Murray-Darling Basin, Australia—Its Future Management* (eds Hart, B. T. et al.) 253–274 (Elsevier, 2020). 10.1016/C2018-0-01363-8.

[52] Post, D. A. et al. Decrease in southeastern Australian water availability linked to ongoing Hadley cell expansion. *Earths Future* **2**, 231–238 (2014).

[53] CSIRO. *Water Availability in the Murray-Darling Basin A Report from CSIRO to the Australian Government*. https://publications.csiro.au/rpr/download?pid=legacy:530&dsid=DS1 (2008).

[54] Hart, B. T. The Australian Murray-Darling Basin Plan: Challenges in its implementation (part 1). *Int. J. Water Resour. Dev.* **32**, 819–834 (2016).

[55] Pittock, J., Williams, J. & Grafton, Q. The Murray-Darling Basin Plan fails to deal adequately with climate change. *Water (Basel)* 26–30 (2015).

[56] Grafton, R. Q. & Wheeler, S. A. Economics of water recovery in the Murray-Darling Basin, Australia. **46**, 55 (2024)

[57] Sheldon, F. *et al.* Are environmental water requirements being met in the Murray–Darling Basin, Australia? *Mar. Freshw. Res.* **75** (2024).

[58] Connell, D. & Grafton, R. Q. Water reform in the Murray-Darling Basin. *Water Resour. Res.* **47** (2011).

[59] Pahl-Wostl, C. Transitions towards adaptive management of water facing climate and global change. *Water Resour. Manag.* **21**, 49–62 (2007).

[60] Kingsford, R. T., Biggs, H. C. & Pollard, S. R. Strategic adaptive management in freshwater protected areas and their rivers. *Biol. Conserv.* **144**, 1194–1203 (2011).

[61] Pittock, J. & Finlayson, C. M. Australia’s MurrayDarling Basin: Freshwater ecosystem conservation options in an era of climate change. *Mar. Freshw. Res.* **62**, 232–243 (2011).

[62] Commonwealth Environmental Water Holder. *Water Management Plan 2023–24*. https://www.dcceew.gov.au/sites/default/files/documents/cewh-water-mgt-plan-2023-24-full.pdf (2023).

[63] Tulbure, M. G., Broich, M., Stehman, S. V. & Kommareddy, A. Surface water extent dynamics from three decades of seasonally continuous Landsat time series at subcontinental scale in a semi-arid region. *Remote Sens. Environ.* **178**, 142–157 (2016).

[64] Heimhuber, V., Tulbure, M. G. & Broich, M. Modeling 25 years of spatio-temporal surface water and inundation dynamics on large river basin scale using time series of Earth observation data. *Hydrol. Earth Syst. Sci.* **20**, 2227–2250 (2016).

[65] Heimhuber, V., Tulbure, M. G. & Broich, M. Modeling multidecadal surface water inundation dynamics and key drivers on large river basin scale using multiple time series of Earth-observation and river flow data. *Water Resour. Res.* **53**, 1251–1269 (2017).

[66] Pekel, J. F., Cottam, A., Gorelick, N. & Belward, A. S. High-resolution mapping of global surface water and its long-term changes. *Nature* **540**(7633), 418–422 (2016). 27926733 10.1038/nature20584

[67] Senanayake, I. P., Yeo, I.-Y. & Kuczera, G. A. Three decades of inundation dynamics in an Australian dryland wetland: An eco-hydrological perspective. *Remote Sens.* **16**, 3310 (2024).

[68] Ceola, S., Laio, F. & Montanari, A. Human-impacted waters: New perspectives from global high-resolution monitoring. *Water Resour. Res.* **51**, 7064–7079 (2015).

[69] Teng, J. *et al.* Two-monthly maximum flood water depth spatial timeseries for the MDB. *CSIRO. Data Collection* (2023). 10.25919/c5ab-h019.

[70] Penton, D. J. et al. The floodplain inundation history of the Murray-Darling Basin through two-monthly maximum water depth maps. *Sci. Data* **10**, 652 (2023). 37741870 10.1038/s41597-023-02559-4 PMC10517945

[71] Ticehurst, C., Teng, J. & Sengupta, A. Development of a multi-index method based on Landsat reflectance data to map open water in a complex environment. *Remote Sens. (Basel)* **14** (2022).

[72] Cohen, S. et al. The Floodwater Depth Estimation Tool (FwDET v2.0) for improved remote sensing analysis of coastal flooding. *Nat. Hazards Earth Syst. Sci.* **19**, 2053–2065 (2019).

[73] Teng, J. *et al.* A comprehensive assessment of floodwater depth estimation models in semiarid regions. *Water Resour. Res.* **58** (2022).

[74] Marvanek, S. *et al.* LIDAR enhanced SRTM Digital Elevation Model (DEM) for Murray Darling Basin. *CSIRO. Data Collection* (2022)

[75] Jeffrey, S. J., Carter, J. O., Moodie, K. B. & Beswick, A. R. Using spatial interpolation to construct a comprehensive archive of Australian climate data. *Environ. Model. Softw.* **16**, 309–330 (2001).

[76] Chiew, F. H. S. & McMahon, T. A. The applicability of morton and penman evapotranspiration estimates in rainfall-runoff modeling. *Water Resour. Bull.* **27**, 611–620 (1991).

[77] Morton, F. I. Operational estimates of areal evapo-transpiration and their significance to the science and practice of hydrology. *J. Hydrol. (Amst.)* **66**, 1–76 (1983).

[78] Chiew, F. H. S. *et al.* Estimating climate change impact on runoff across southeast Australia: Method, results, and implications of the modeling method. *Water Resour. Res.* **45** (2009).

[79] Zheng, H. et al. Projections of future streamflow for Australia informed by CMIP6 and previous generations of global climate models. *J. Hydrol. (Amst.)* **636**, 131286 (2024).

[80] Perrin, C., Michel, C. & Andreassian, V. Improvement of a parsimonious model for streamflow simulation. *J. Hydrol. (Amst.)* **279**, 275–289 (2003).

[81] Chiew, F. H. S. *et al.* Future runoff projections for Australia and science challenges in producing next generation projections. 1745–1751 Preprint at http://www.mssanz.org.au/modsim2017/L16/chiew.pdf (2017).

[82] Zheng, H., Chiew, F. H. S., Potter, N. J. & Kirono, D. G. C. Projections of water futures for Australia: an update. 1000–1006 Preprint at https://mssanz.org.au/modsim2019/K7/zhengH.pdf (2019).

[83] Viney, N. R. *et al.* The usefulness of bias constraints in model calibration for regionalisation to ungauged catchments. In *18th World IMACS Congress and MODSIM09 International Congress on Modelling and Simulation* 3421–3427 Preprint at http://www.mssanz.org.au/modsim09/I7/viney_I7a.pdf (2009).

[84] Blöschl, G. et al. Twenty-three unsolved problems in hydrology (UPH)—A community perspective. *Hydrol. Sci. J.* **64**, 1141–1158 (2019).

[85] Fowler, K. J. A., Peel, M. C., Western, A. W., Zhang, L. & Peterson, T. J. Simulating runoff under changing climatic conditions: Revisiting an apparent deficiency of conceptual rainfall-runoff models. *Water Resour. Res.* **52**, 1820–1846 (2016).

[86] Saft, M., Peel, M. C., Western, A. W., Perraud, J. M. & Zhang, L. Bias in streamflow projections due to climate-induced shifts in catchment response. *Geophys. Res. Lett.* **43**, 1574–1581 (2016).

