# Supplementary material for: Floodplain inundation in the Murray–Darling Basin under current and future climate conditions
Source: Sci Rep. 2025 Mar 14;15:8917. doi: 10.1038/s41598-025-93670-6 (PMC11909220; doi:10.1038/s41598-025-93670-6)
Supplement: Supplementary file 1 — Supplementary Information. [file 41598_2025_93670_MOESM1_ESM.docx]

Floodplain inundation in the Murray-Darling Basin under current and future climates

Jin Teng^1^*[
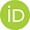
](https://orcid.org/0000-0002-0045-1448), Francis H.S. Chiew^1^[
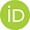
](http://orcid.org/0000-0001-8020-8773), Hongxing Zheng^1^[
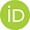
](https://orcid.org/0000-0001-6410-8326), Ang Yang^1^, David J. Penton^1^[
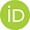
](https://orcid.org/0000-0002-4444-9761), Catherine Ticehurst^1^[
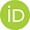
](https://orcid.org/0000-0001-8567-1388), Steve Marvanek^1^[
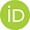
](https://orcid.org/0000-0001-9162-0923), Jai Vaze^1^, Fathaha Khanam^2^, David A. Post^1^, Carmel Pollino^1^

^1^ Commonwealth Scientific and Industrial Research Organization; Canberra, 2601, Australia.

^2^ Murray-Darling Basin Authority; Canberra, 2601, Australia.

*Corresponding author: Jin Teng. Email: [jin.teng@csiro.au](mailto:jin.teng@csiro.au)

Supplementary Materials


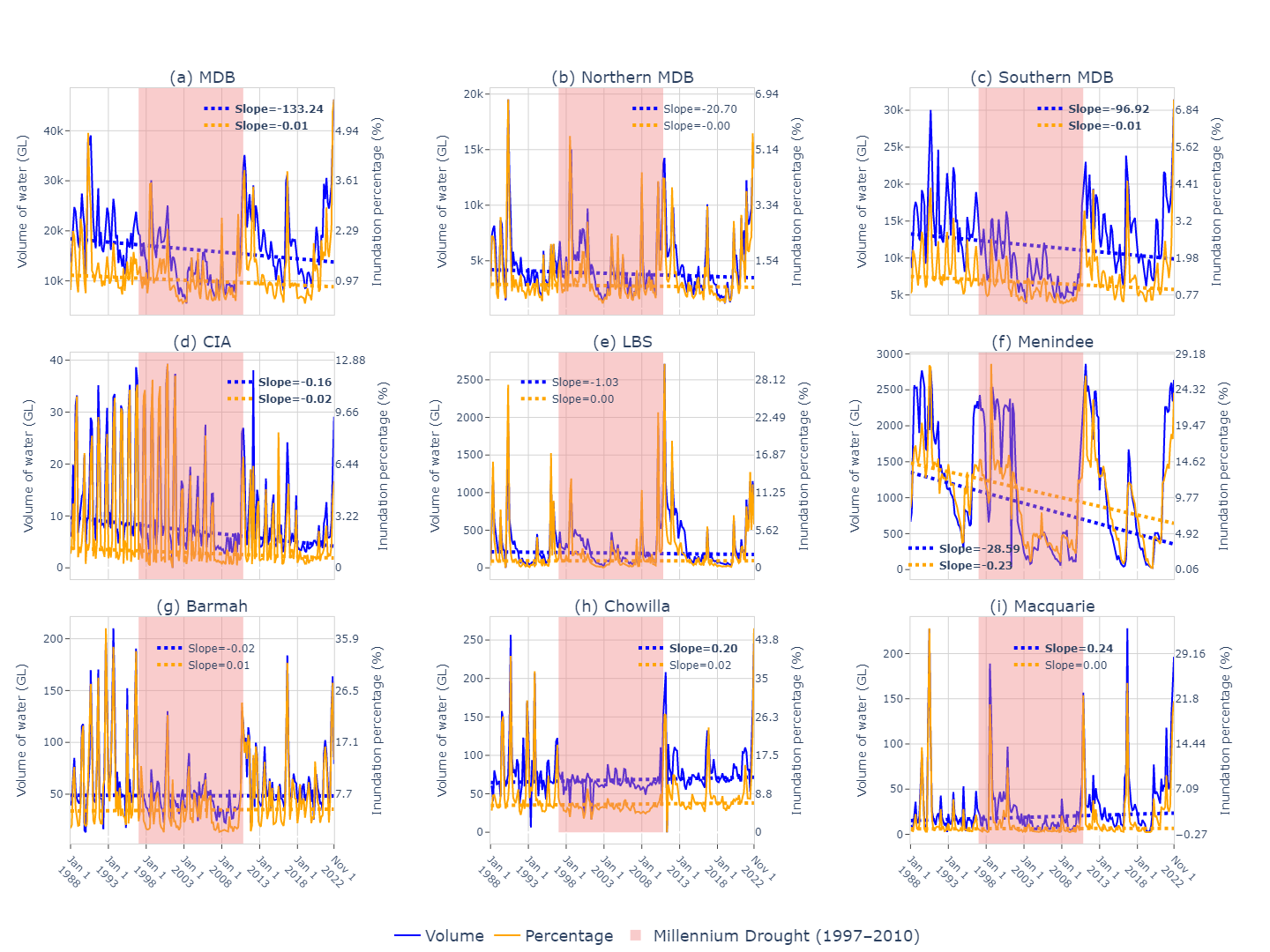


**Figure S1.** **Two monthly maximum flood extent (orange) and volume (blue) for each region.** The trends (GL per two months or % per two months), are shown on the top right and as trendlines in each plot. The bold font indicates that the trend is statistically significant at α < 0.05.

**Table S1** Intra-annual variability (MV), interannual variability (CV) and trends of floodplain inundation in the Murray-Darling Basin. Area (A) and Vol (V) denotes area of inundation extent (km^2^) and inundation water volume (GL) respectively. The subscription ‘max’ and ‘min’ means maximum and minimum values of the variables in a year respectively.

| Regions | MV | | CV | | | | Trend (in GL/a or km^2^/a) | | | | Trend (in percentage/a) | | | |
| --- | --- | --- | --- | --- | --- | --- | --- | --- | --- | --- | --- | --- | --- | --- |
|  | Vol. | Area | V_max_ | V_min_ | A_max_ | A_min_ | V_max_ | V_min_ | A_max_ | A_min_ | V_max_ | V_min_ | A_max_ | A_min_ |
| MDB | 1.79 | 2.85 | 0.39 | 0.35 | 0.64 | 0.38 | -55.9 | -19.7 | 76.8 | -1.97 | -0.25 | -0.16 | 0.36 | -0.03 |
| North Basin | 2.75 | 4.81 | 0.57 | 0.51 | 0.76 | 0.65 | -9.74 | 16.2 | 26.9 | 43.3 | -0.13 | 0.55 | 0.21 | 1.56 |
| South Basin | 1.84 | 2.46 | 0.38 | 0.35 | 0.64 | 0.34 | -80.7 | -24.2 | 14.2 | -36.4 | -0.50 | -0.27 | 0.13 | -0.86 |
| CIA | 5.24 | 19.3 | 0.50 | 0.31 | 0.56 | 0.43 | -0.68 | -0.07 | -52.3 | -1.03 | -3.04 | -1.46 | -3.80 | -1.24 |
| LBS | 3.62 | 8.42 | 1.00 | 0.90 | 1.18 | 1.24 | 5.09 | 1.84 | 1.29 | 0.72 | 0.88 | 1.10 | 0.96 | 3.83 |
| Menindee | 5.63 | 2.95 | 0.66 | 0.98 | 0.56 | 0.69 | -28.2 | -18.5 | -7.49 | -6.81 | -1.91 | -2.48 | -1.62 | -2.74 |
| Barmah | 3.49 | 7.79 | 0.51 | 0.32 | 0.61 | 0.46 | -1.55 | 0.20 | -3.70 | -0.10 | -1.56 | 0.58 | -1.71 | -0.33 |
| Chowilla | 2.93 | 2.49 | 0.49 | 0.28 | 0.81 | 0.22 | -0.72 | 0.80 | -0.85 | 0.23 | -0.69 | 1.53 | -1.16 | 0.80 |
| Macquarie | 7.14 | 25.8 | 0.98 | 0.86 | 1.37 | 1.35 | 0.54 | 0.46 | -0.58 | 0.99 | 0.85 | 3.56 | -0.22 | 5.22 |

**Table S2.** Coefficient of determination (R²) between annual maximum flood extent and rainfall/runoff metrics. For the six irrigation and ecological sites, the hydroclimate variables were aggregated over their upstream contributing areas. The two highest values for each region are denoted in bold font.

| Rainfall/runoff metrics | MDB | Northern MDB | Southern MDB | CIA | LBS | Menindee | Barmah | Chowilla | Macquarie |
| --- | --- | --- | --- | --- | --- | --- | --- | --- | --- |
| Annual rainfall | 0.58 | 0.54 | 0.57 | **0.07** | 0.37 | 0.22 | 0.34 | 0.42 | 0.36 |
| Maximum 7-day rainfall | 0.32 | 0.26 | 0.2 | 0.04 | 0.23 | 0.11 | 0 | 0.27 | 0.15 |
| Maximum 30-day rainfall | 0.18 | 0.21 | 0.08 | 0.05 | 0.16 | 0.02 | 0.05 | 0.18 | 0.16 |
| Annual runoff | 0.82 | **0.77** | **0.84** | 0.06 | **0.71** | 0.52 | **0.69** | **0.61** | **0.84** |
| Maximum 7-day runoff | **0.85** | 0.68 | 0.77 | 0.06 | 0.7 | **0.55** | 0.59 | 0.51 | 0.77 |
| Maximum 30-day runoff | **0.91** | **0.84** | **0.92** | **0.07** | **0.9** | **0.62** | **0.69** | **0.52** | **0.82** |
